# CircFAM120B knockdown inhibits osteosarcoma tumorigenesis via the miR-1205/PTBP1 axis

**DOI:** 10.18632/aging.203657

**Published:** 2021-10-29

**Authors:** Jia-Ju Li, Ming-Yue Xiong, Tian-Yu Sun, Chang-Bin Ji, Run-Tao Guo, Ya-Wei Li, Hong-Yu Guo

**Affiliations:** 1 Department of Traumatology, The First Affiliated Hospital, And College of Clinical Medicine of Henan University of Science and Technology, Luoyang 471003, Henan, China; 2 Department of Ophthalmology, XiPing County People’s Hospital, Zhumadian 463900, Henan, China

**Keywords:** circRNA, os

## Abstract

Background: Osteosarcoma (OS) is a highly prevalent bone malignancy with poor clinical outcomes. Expression of the circular RNA, hsa_circ_0078767 (circFAM120B) is elevated in OS, however, its mechanisms in OS are unclear.

Methods: CircFAM120B levels were detected in OS tissue and cell lines. Silenced circFAM120B experiments were performed to assess its effects on OS *in vitro* cancer phenotypes and *in vivo* tumor growth. Then, bioinformatics analyses were used to predict circFAM120B target microRNAs (miRNAs) and associated genes.

Results: CircFAM120B and the transcription factor, PTBP1 were elevated in OS tissue and cell lines, while miR-1205 was poorly expressed. Silenced circFAM120B significantly suppressed *in vitro* OS cell proliferation and invasion, and inhibited *in vivo* tumor growth. CircFAM120B also appeared to function as an miR-1205 sponge, as miR-1205 bound to PTBP1. Interestingly, overexpressed PTBP1 (or miR-1205 inhibition) reversed the inhibitory effects mediated by circFAM120B downregulation in OS cells.

Conclusion: We hypothesize circFAM120B functions as a miR-1205 sponge to elevate PTBP1 levels, enhancing OS progression and associated malignant phenotypes. Thus, circFAM120B may function as a crucial mediator during OS progression.

## INTRODUCTION

Osteosarcoma (OS) is a severe bone malignancy in young people, with elevated local invasion and distant metastasis properties [1, 2]. Despite developments in multidisciplinary therapies, the high incidence of metastasis makes a poor survival in OS patients [3, 4]. Therefore, a better understanding of the biological mechanisms underpinning OS will help scientists develop biomarker-targeted therapies optimized for OS patients.

Circular RNAs (circRNAs) are generated by the non-canonical back splicing of pre-messenger RNA, where structures are typified by a closed loop out of the 5’ or 3’ free polarity [5, 6]. Dysregulated circRNAs are closely associated with several key cancer phenotypes during cancer progression [7]. CircRNA_069718 promoted key cancer phenotypes in triple-negative breast cancer via Wnt/β-catenin signaling [8]. CircRNA_102171 overexpression stimulated papillary thyroid cancer progression by activating Wnt/β-catenin signaling in a CTNNBIP1-dependent manner [9]. Equally, hsa_circ_0001361 stimulated bladder cancer phenotypes via miR-491-5p/MMP9 signaling [10] and similarly, hsa_circ_0078767 (circFAM120B) was elevated in OS [11]. However, definitive circFAM120B mechanisms in OS remain unknown.

Mechanistically, circRNAs act as “sponges” of microRNAs (miRNAs) to alter miRNAs actions and subsequent downstream targets [12]. CircSERPINA3 promoted key cancer phenotypes by sponging miR-944 and altering MDM2 levels during nasopharyngeal carcinoma [13]. Also, hsa_circ_0007494 suppressed prostate cancer by sponging miR-616 to enhance PTEN levels [14]. Additionally, circMYO10 stimulated OS by sponging miR-370-3p and altering RUVBL1 levels to promote β-catenin/LEF1 transcriptional activity via chromatin remodeling effects [15]. However, relationships between circFAM120B, miR-1205, and PTBP1 in OS are unclear and require investigation.

Here, circFAM120B expression was highly elevated in OS. Moreover, by sponging miR-1205, circFAM120B promoted PTBP1 expression and increased OS proliferation and invasion, suggesting a mechanistic function for circFAM120B in OS.

## MATERIALS AND METHODS

### Participants and samples

Our research was ethically approved by the Ethics Committee of The First Affiliated Hospital and College of Clinical Medicine of Henan University of Science and Technology. All protocols conformed to ethical principles governing both human and animal subjects as indicated by national and international guidelines. Tissue samples from 37 patients with OS (paired normal and primary tumor tissues) were collected from The First Affiliated Hospital and College of Clinical Medicine of Henan University of Science and Technology. All participants provided written informed consent. Resected or biopsy samples were rapidly frozen in liquid nitrogen and stored at -80° C.

### Cell growth conditions

Human OS cell lines (MG63, U2OS, 143B, HOS, and SJSA-1) and osteoblasts, hFOB 1.19 were supplied by the Chinese Academy of Sciences. Cultures were maintained in Dulbecco’s modified Eagle medium (DMEM) (Thermo Fisher Scientific, Waltham, MA, USA) supplemented with 10% fetal bovine serum (FBS) (Invitrogen, Waltham, MA, USA) and penicillin (100 U/mL) at 37° C in 5% CO_2_.

Small interfering RNA (siRNA) molecules for circFAM120B (si-circFAM120B#1: 5’-ATGACCATTCCAGGTGAACGA-3’, si-circFAM120B#2: 5’-CAGATGACCATTCCAGGTGAA-3’, si-circFAM120B#3: 5’- AGATGACCATTCCAGGTGAAC-3’), miR-1205 mimics, miR-1205 inhibitors, PTBP1 overexpression plasmids, and corresponding negative controls were generated by GenePharma (Shanghai, China). Lipofectamine™3000 (Invitrogen) was used for transfections.

### Quantitative real-time PCR (qRT-PCR)

TRIzol reagent (Invitrogen) was used to isolate RNA from all samples based on manufacturer’s instructions. The PowerUp™ SYBR™ green master mix (Applied Biosystems, Foster City, CA, USA) was used to determine circFAM120B, miR-1205, and PTBP1 levels. The QuantStudio RT-PCR system (Applied Biosystems) was used to conduct qRT-PCR. Using 2^-ΔΔCt^, relative target expression and U6 and GAPDH internal controls were determined. The following primers were used: circFAM120BFor, 5’-ACCTGCCTAGCTGTCAAGGA-3’, Rev, 5’GGATCTAGAGATGCGCCAAC-3’; miR-1205For, 5’-CTGCAGGGTTTGCTTTGAGG-3’, Rev, 5’CTCCAGAACAGGGTTGACAGG-3’; PTBP1For, 5’CTGAGGATCCATGTCTGGTTATTCTAGTG-3’, Rev, 5’-TTACTCTCGAGTTACTGGGAATATCCGGTT-3’.

### Cell Counting Kit-8 (CCK-8) assay

Using the Cell Counting Kit-8 kit (CCK-8, Beyotime, Shanghai, China), cell viability was examined. Briefly, transfected OS cells at 2×10^4^ cells/well were incubated for 1, 2 or 3 days. Then, 10 μL CCK-8 reagent/well was added for 2 h and absorbance at 450 nm was measured on a microplate reader (Life Science, Hercules, CA, USA).

### Colony formation

Approximately 1 × 10^3^ transfected OS cells/well were grown in 6-well plates for 2 weeks. Colonies were fixed in 4% paraformaldehyde (Sigma-Aldrich, St. Louis, MO, USA) for 5 min and stained with 1% crystal violet (Sigma-Aldrich) for 60 s. Colony images were captured by a microscope (Nikon, Japan).

### The 5-ethynyl-2'-deoxyuridine (EdU) assay

Transfected OS cells in 96-well plates (2 × 10^3^ cells/well) were cultivated for 48 h. Then, EdU solution was diluted to 50 μM in cell medium and added. Plates were incubated for 120 min at 37° C, fixed in formaldehyde, stained with Apollo, and examined under an inverted fluorescence microscope (Nikon, Japan).

### Transwell invasion assay

Transwell assays determined OS cell invasion capabilities. Chambers were coated in matrigel (Corning, Midland, Michigan, USA). Then, 200 μL cells in serum-free medium were added to upper chambers and 600 μL complete medium to lower chambers. After 2 days, invading cells were fixed with methanol, stained with 0.1% crystal violet, and photographed and recorded using microscopy (Nikon, Japan).

### Dual-luciferase assay

Partial circFAM120B sequences and the PTBP1 3’-untranslated region (UTR) containing miR-1205 sites were generated by GenePharma and cloned into pmirGLO vectors to generate wild-type (WT) or mutant (WT) reporter circFAM120B and PTBP1 plasmids. Plasmids were then transfected into cells plus miR-1205 agomir reagents. Results were quantified and normalized to Renilla luciferase activities (Beyotime).

### RNA pull-down assay

RNA plasmids were constructed by RiboBio (Shanghai, China). RNA was marked with a biotin probe and co-incubated with cell lysates to form probe-coated beads. After this, bead bound RNA complexes were processed using a RNeasy mini kit (Qiagen) for expression analyses [16].

### Western blotting assay

Protein from OS cells was extracted using RIPA buffer (Keygen Biotech, Shanghai, China) and quantitatively assayed using the bicinchoninic acid protein quantification kit. Proteins were electrophoresed on sodium dodecyl sulfate-polyacrylamide gels, transferred to polyvinylidene fluoride membranes, and blocked for 60 min in 5% fat-free milk. Then, primary antibodies were added overnight at 4° C, membranes washed in TBST, and further incubated with corresponding secondary antibodies. Then, proteins were visualized using an enhanced chemiluminescent (ECL, Thermo Fisher Scientific) detection system.

### *In vivo* xenograft model

For *in vivo* tumorigenesis assays, male BALB/c nude mice (1 month old) were subcutaneously injected with 1×10^7^ OS cells expressing sh-circFAM120B or sh-NC plasmids. Palpable tumors were measured weekly. After 6 weeks, tumors were removed and weights recorded. CircFAM120B, miR-1205, and PTBP1 levels were also quantitated by RT-PCR. The animal study has been approved by the ethical committee of The First Affiliated Hospital, and College of Clinical Medicine of Henan University of Science and Technology.

### Statistical analyses

SPSS 18.0 was used for statistical analyses and data was expressed as the mean ± standard deviation (SD). Student’s t-tests were used to analyze differences between two groups and one-way analysis of variance (ANOVA) followed by Tukey’s *post hoc* test were used to determine differences between multiple groups. A P < 0.05 was statistically significant.

### Ethics approval and consent to participate

Ethical approval was given by the Ethics Committee of The First Affiliated Hospital, and College of Clinical Medicine of Henan University of Science and Technology.

### Availability of data and materials

The dataset supporting the conclusions of this article is included within the article.

## RESULTS

### Aberrant circFAM120B elevation during OS

**Figure 1:**
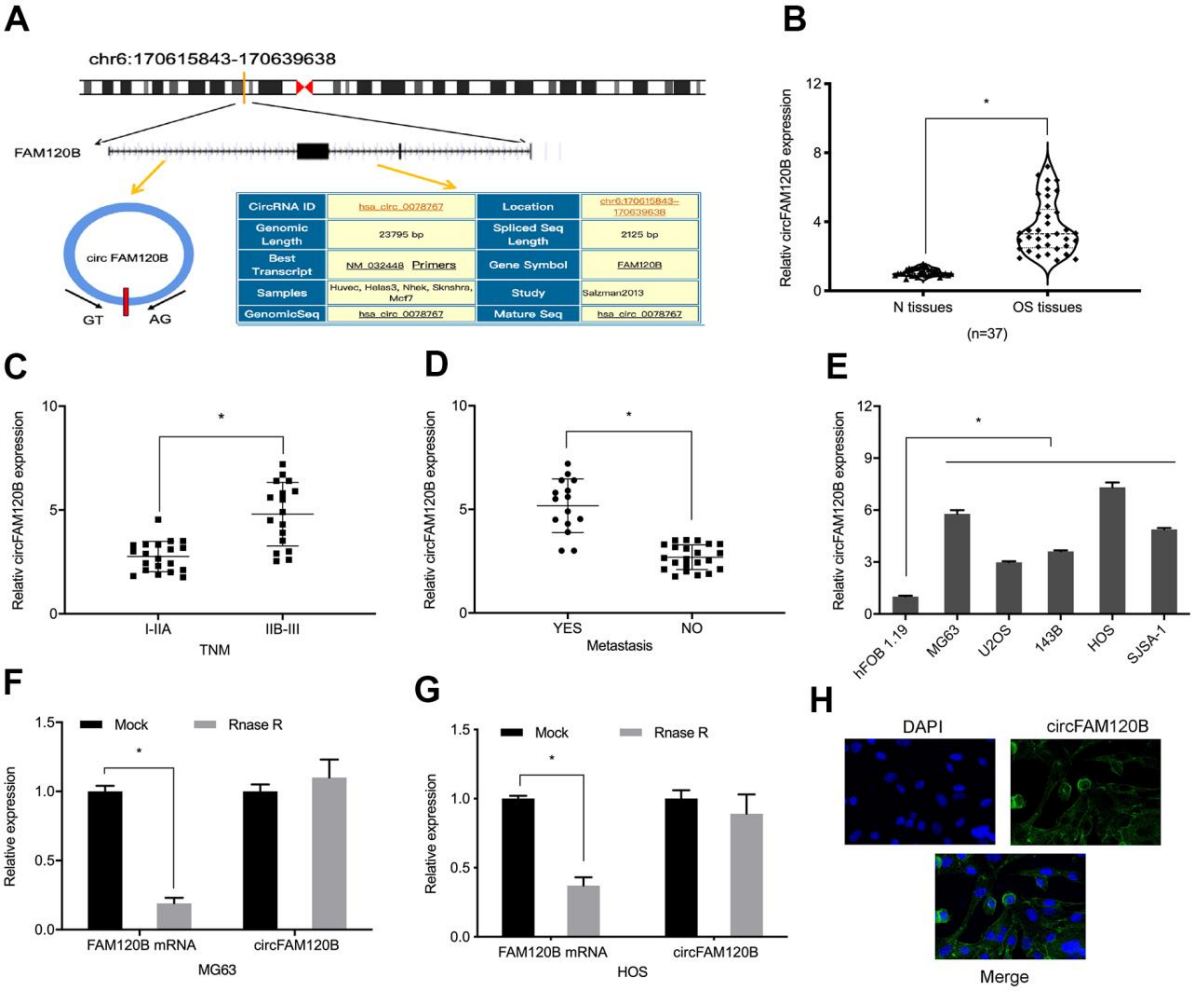
**Aberrant circFAM120B up-regulation in OS.** (**A**) CircFAM120B schematic. (**B**) Relative circFAM120B levels in OS tissues. (**C**, **D**) Elevated circFAM120B levels were associated with OS advanced TNM stage and metastasis. (**E**) Relative circFAM120B levels in (**H**) CircFAM120B localized mainly to the cytoplasm of OS cells lines. (**F**) circFAM120B mRNA exhibited higher resistance to RNase R digestion. (**G**) CircFAM120B localized to the cytoplasm of OS cells. *P < 0.05.

Previously, hsa_circ_0078767 (circFAM120B) (Figure 1A) was reportedly elevated in four OS tissues relative to paired non-tumor tissues [11]. To confirm these data, we investigated circFAM120B relative expression in our 37 OS samples. Our expression analyses showed that circFAM120B levels were significantly elevated and were related to advanced TNM grade and metastasis in patients with OS (Figure 1B–1D). Also, OS cell lines had higher circFAM120B levels in comparison to hFOB 1.19 cells (Figure 1E). When compared with FAM120B mRNA expression, circFAM120B mRNA exhibited higher resistance to RNase R digestion (Figure 1F, 1A, 1G) suggesting circFAM120B mRNA was more stable than linear RNA. Moreover, FISH assay showed that circFAM120B localized mainly to the cytoplasm of OS cells (Figure 1H).

**Figure 2:**
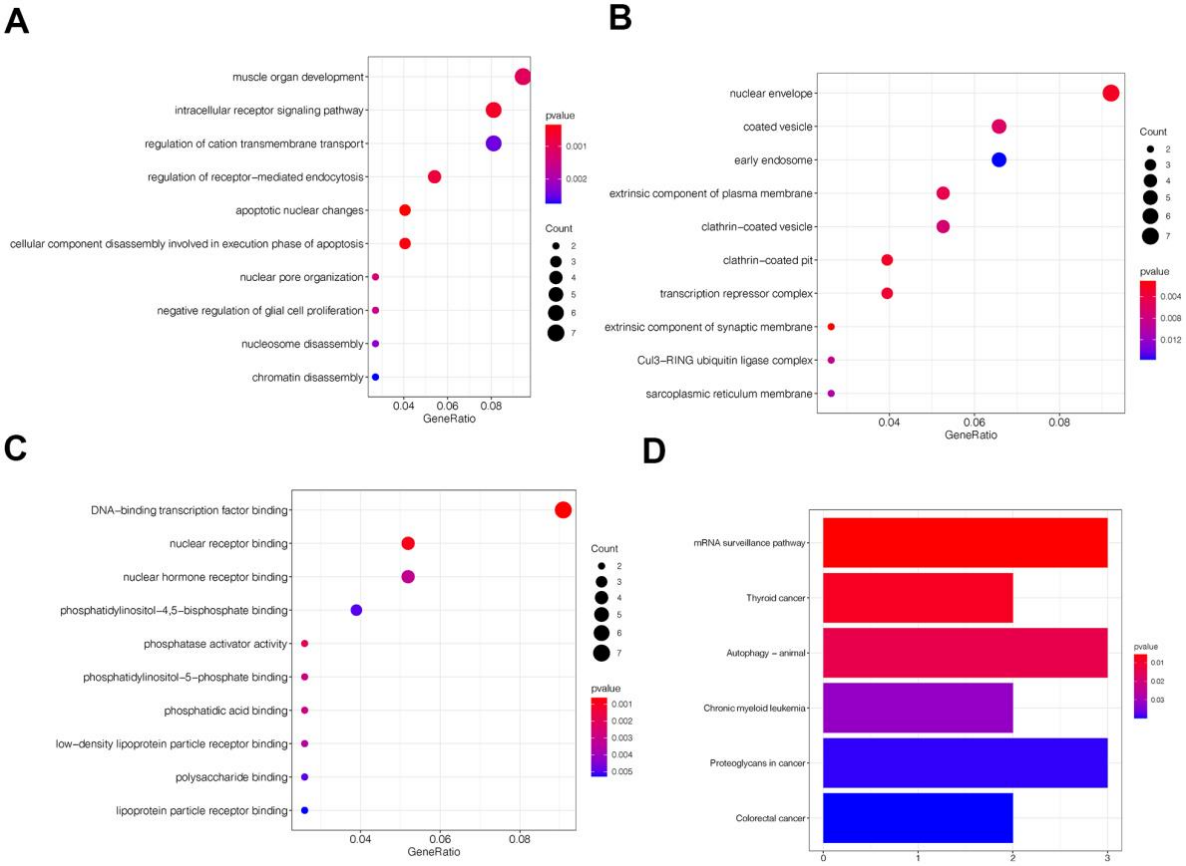
**Gene ontology (GO) and Kyoto encyclopedia and genes and genomes (KEGG) analyses of circFAM120B in OS.** (**A**–**C**) Top 10 circFAM120B enriched terms from GO analyses. (**D**) Top 5 circFAM120B enriched pathways from KEEG analyses.

Gene ontology (GO) and Kyoto Encyclopedia of Genes and Genomes (KEGG) pathway analyses indicated circFAM120B was related to cell motility (Figure 2A–2C) and tumor progression, respectively (Figure 2D).

### circFAM120B suppression inhibited OS cells proliferation and invasion

**Figure 3:**
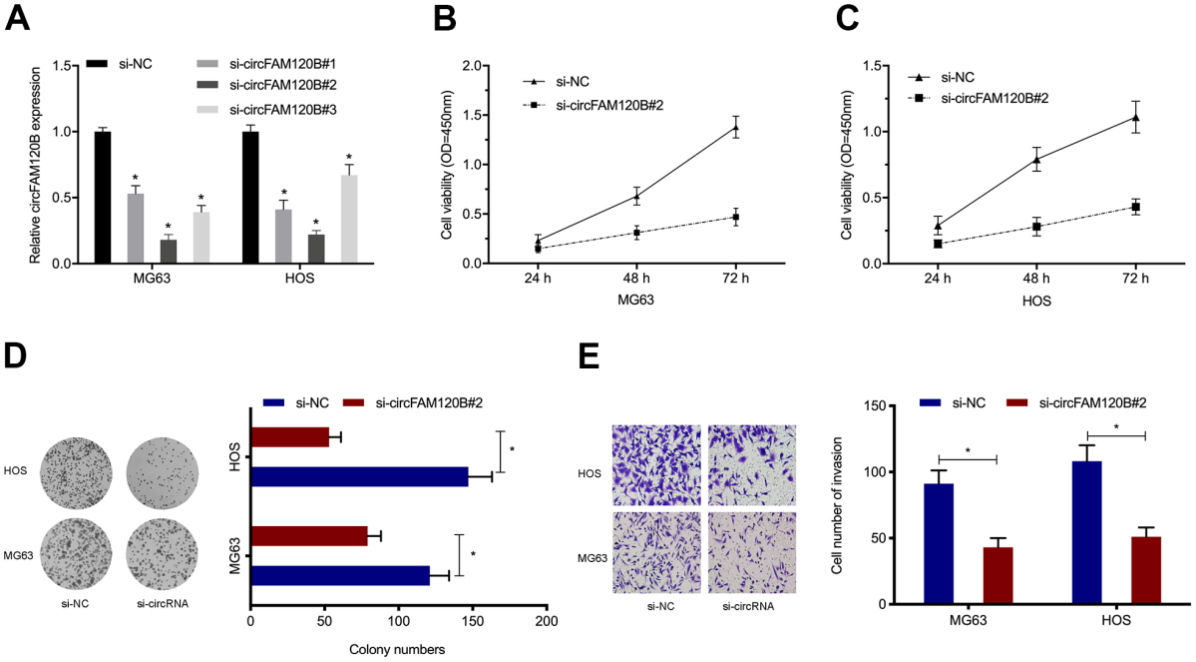
**circFAM120B promotes OS cell proliferation and invasion *in vitro*.** (**A**) OS cells expressing si-circFAM120B and si-NC was determined by expression analysis. (**B**–**D**) OS cells viability was explored by CCK-8 and colony formation assays. (**E**) OS cells invasion ability was explored by transwell assays. *P < 0.05.

To examine circFAM120B functions, we constructed three siRNA oligonucleotides to silence circFAM120B and then assessed knockdown efficiency in OS cells (Figure 3A). CCK-8 assays showed circFAM120B silencing suppressed OS viability (Figure 3B, 3C). Similarly, colony formation assays showed that downregulated circFAM120B levels inhibited cell cloning efficiency (Figure 3D). Additionally, cell invasion capabilities after circFAM120B silencing were also significantly decreased (Figure 3E). Combined, circFAM120B promoted OS *in vitro* cell proliferation and invasion capabilities.

### circFAM120B binds miR-1205 in OS

**Figure 4:**
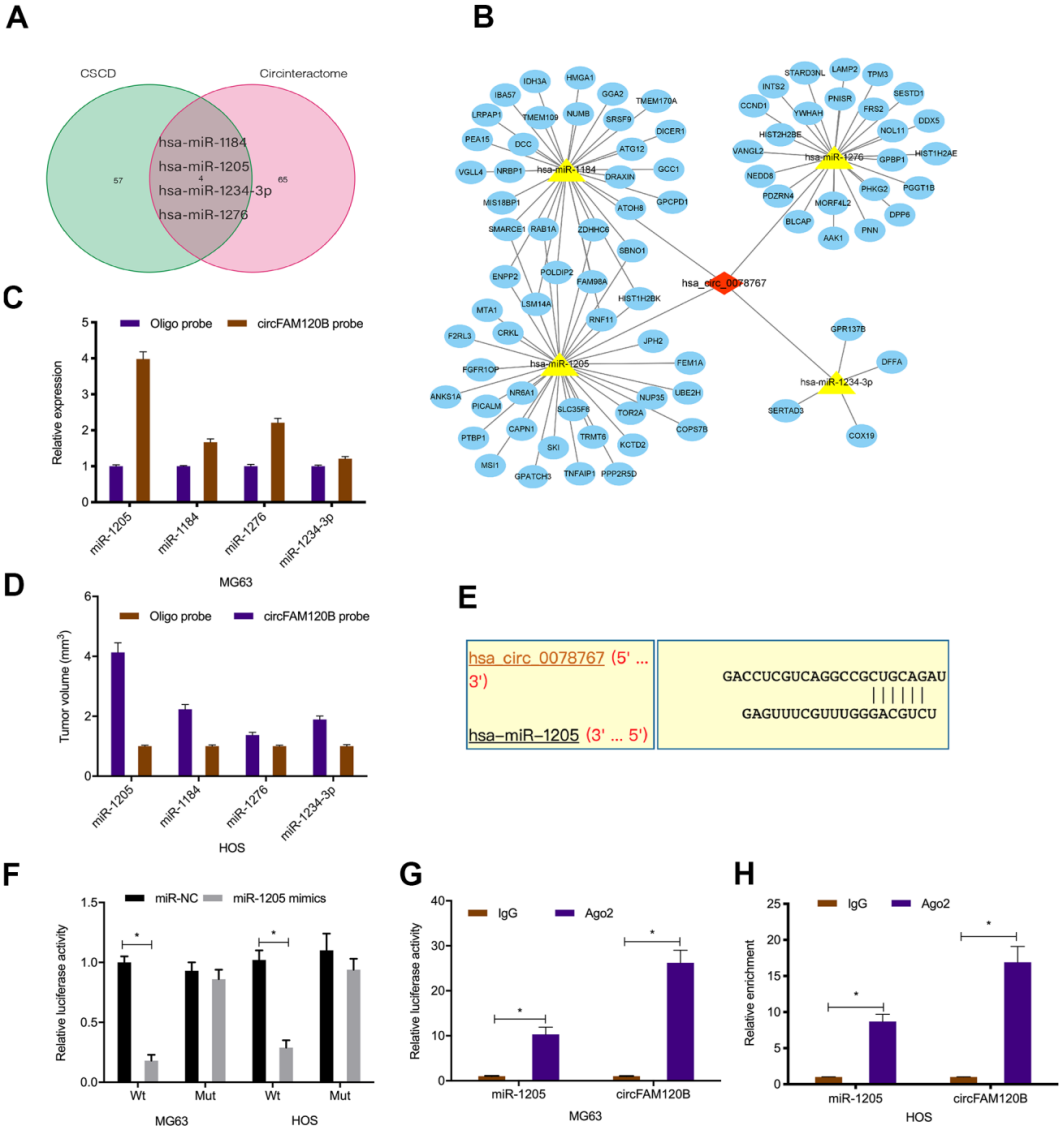
**Interactions between circFAM120B and miR-1205.** (**A**) Schematic of overlapping target miRNAs for circFAM120B. (**B**) Genes, miRNAs, and circFAM120B regulatory network. (**C**, **D**) Relative expression levels of four miRNA candidates in OS cells lysates were detected by qRT-PCR. (**E**) Binding site predictions between circFAM120B and miR-1205. (**F**–**H**) Molecular interactions between circFAM120B and miR-1205 were explored by luciferase reporter assays and RIP assay. *P < 0.05.

circRNAs exert their biological effects by interacting with miRNAs [17, 18]. In this study, circRNA-miRNA-mRNA network analyses revealed that circFAM120B contained binding sites for miR-1205, miR-1184, miR-1276, and miR-1234-3p (Figure 4A, 4B). Pull-down assays showed miR-1205 was highly enriched in RNAs when using the circFAM120B probe in comparison to the oligonucleotide probe (Figure 4C, 4D). Luciferase reporter assay data showed that activities in OS cells expressing circFAM120B-WT and miR-1205 mimics were inhibited (Figure 4E, 4F). RIP assay data confirmed circFAM120B and miR-1205 were enriched in AGO2 cell line immunoprecipitants (Figure 4G, 4H). Combined, circFAM120B appeared to function as a sponge for miR-1205.

### MiR-1205 suppresses OS cells proliferation and invasion

**Figure 5:**
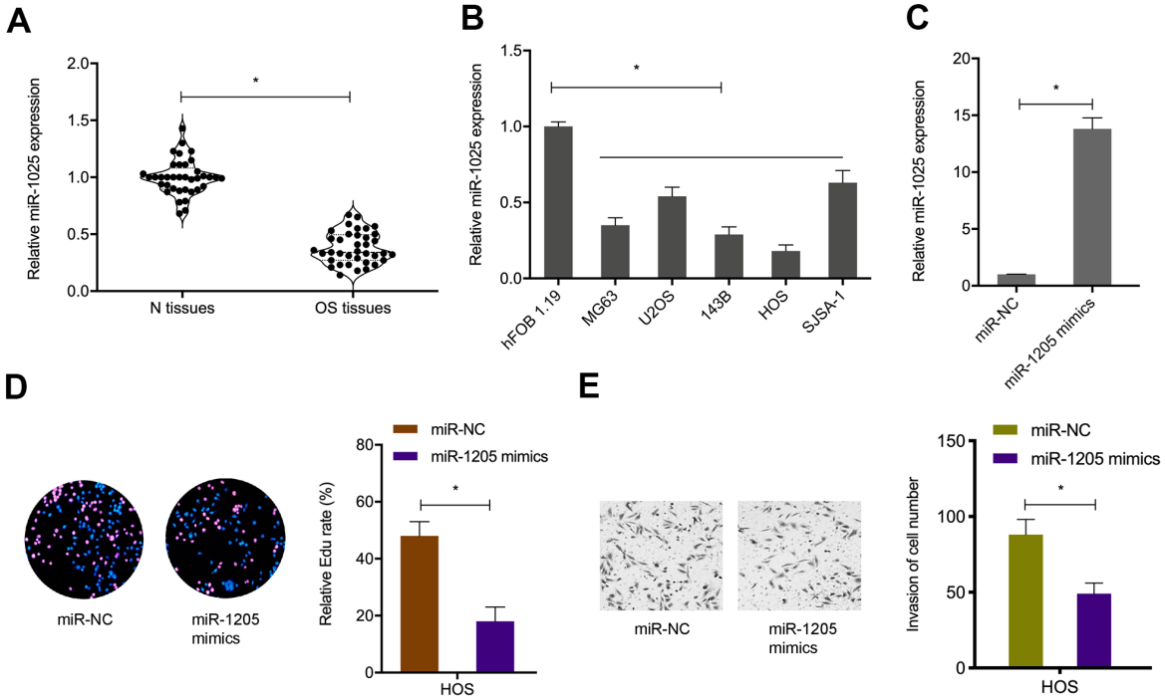
**MiR-1205 suppresses OS progression.** (**A**, **B**) MiR-1205 levels in OS samples and cell lines. (**C**) Transfection efficiencies of miR-1205 mimics in HOS cell line. (**D**, **E**) MiR-1205 overexpression reduces HOS cells proliferation and invasion. *P < 0.05.

To ascertain mIR-1205 functional insights, miR-1205 levels in OS samples and cell lines were explored (Figure 5A, 5B). Next, miR-1205 mimics were transfected into OS cells (Figure 5C), Edu proliferation assays showed that miR-1205 overexpression inhibited *in vitro* OS cells proliferation (Figure 5D). We also observed similar data for miR-1205 effects on *in vitro* OS cells invasion (Figure 5E). Collectively, miR-1205 putatively functioned as a tumor suppressor miRNA during OS progression.

### MiR-1205 negatively inhibits PTBP1 expression

**Figure 6:**
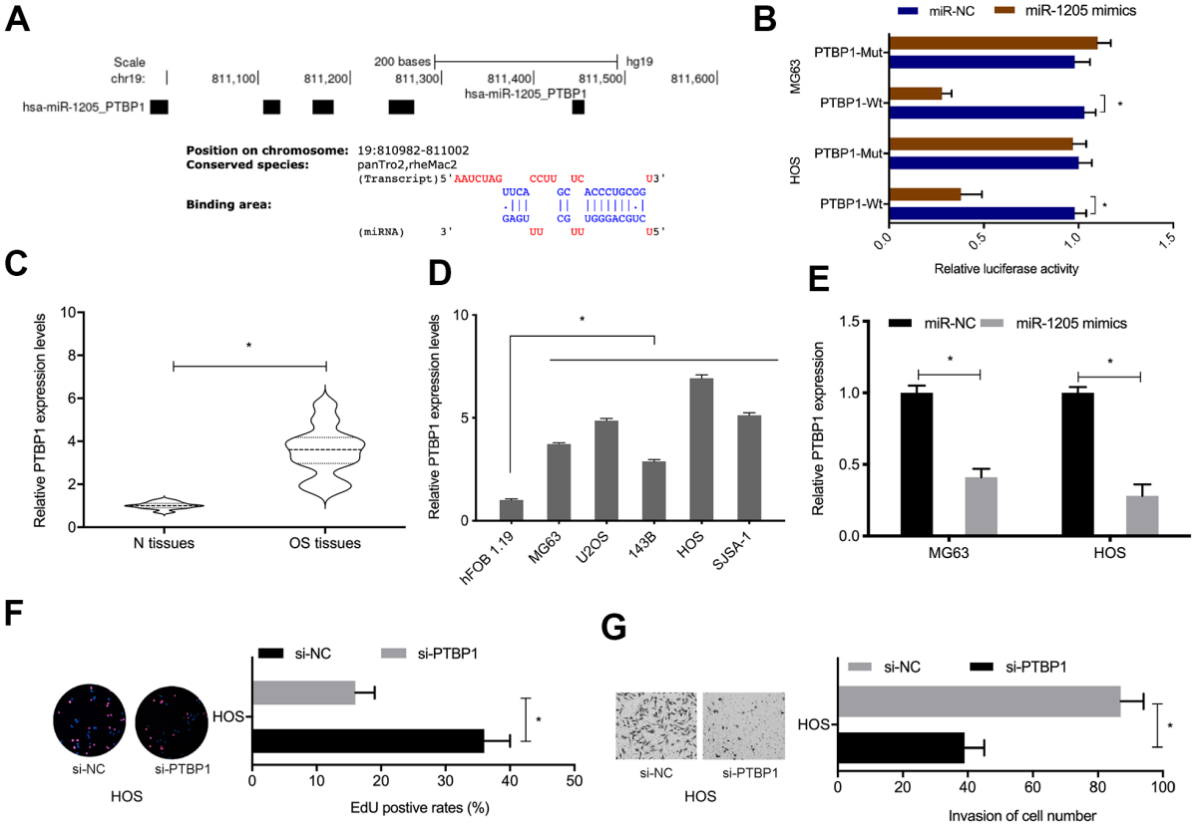
**PTBP1 is directly targeted by miR-1205.** (**A**, **B**) Molecular interactions between miR-1205 and PTBP1. (**C**, **D**) Relative PTBP1 expression in OS tissues and cell lines. (**E**) MiR-1205 overexpression reduces PTBP1 expression in OS cells. (**F**, **G**) Suppressing PTBP1 reduces HOS cells proliferation and invasion. *P < 0.05.

Next, PTBP1 was selected as target of miR-1205 for further investigation (Figure 6A). To confirm the regulatory relationship, luciferase activities were investigated. We observed that miR-1205 overexpression inhibited the luciferase activity of the PTBP1-WT group, while no effects were seen in the mutant group (Figure 6B). qRT-PCR analyses revealed that PTBP1 levels were significantly increased in OS tissues and cell lines (Figure 6C, 6D). MiR-1205 overexpression reduced PTBP1 protein levels in OS cells (Figure 6E). Furthermore, functional assays showed that suppressing PTBP1 reduced HOS cells proliferation an invasion abilities *in vitro* (Figure 6G. 6H). Taken together, PTBP1 appeared to be a direct target of miR-1205.

### circFAM120B promotes OS progression via miR-1205/PTBP1 signaling

**Figure 7:**
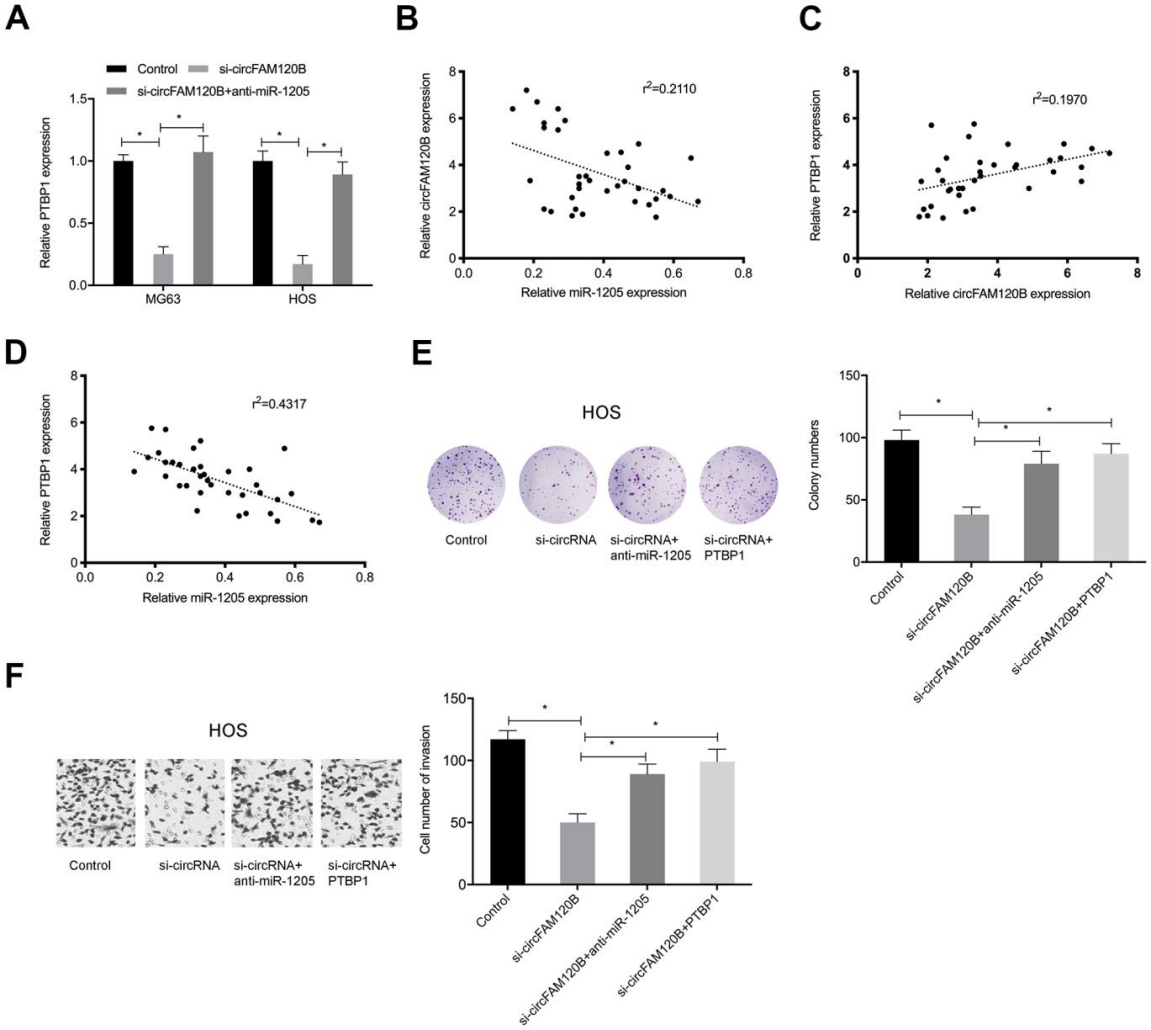
**circFAM120B inhibits OS progression via miR-1205/PTBP1 signaling.** OS cells transfected with si-circFAM120B or co-transfected with miR-1205 inhibitors or co-transfected with PTBP1. (**A**) PTBP1 mRNA levels in OS cells. (**B**–**D**) Expression correlations between circFAM120B, miR-1205, and PTBP1 in OS tissues. (**E**) Colony formation assay. (**F**) Transwell invasion assay. *P < 0.05.

To explore if circFAM120B enhanced OS progression by modulating the miR-1205/PTBP1 signaling pathway, rescue studies were conducted. Expression data showed that suppressing circFAM120B significantly reduced PTBP1 levels in OS cells, while miR-1205 inhibitors abolished these effects (Figure 7A). Correlation analyses revealed that circFAM120B levels were negatively associated with miR-1205 levels but positively associated with PTBP1 in OS tissues (Figure 7B, 7C). MiR-1205 was negatively correlated with PTBP1 levels in OS tissues (Figure 7D). Also, OS cells proliferation and invasion abilities were significantly reduced by suppressing circFAM120B, while miR-1205 inhibitors or PTBP1 overexpression rescued these effects (Figure 7E, 7F). Thus, circFAM120B promoted cell proliferation and invasion by regulating miR-1205/PTBP1 signaling during OS.

### circFAM120B promotes OS cells growth *in vivo*

**Figure 8:**
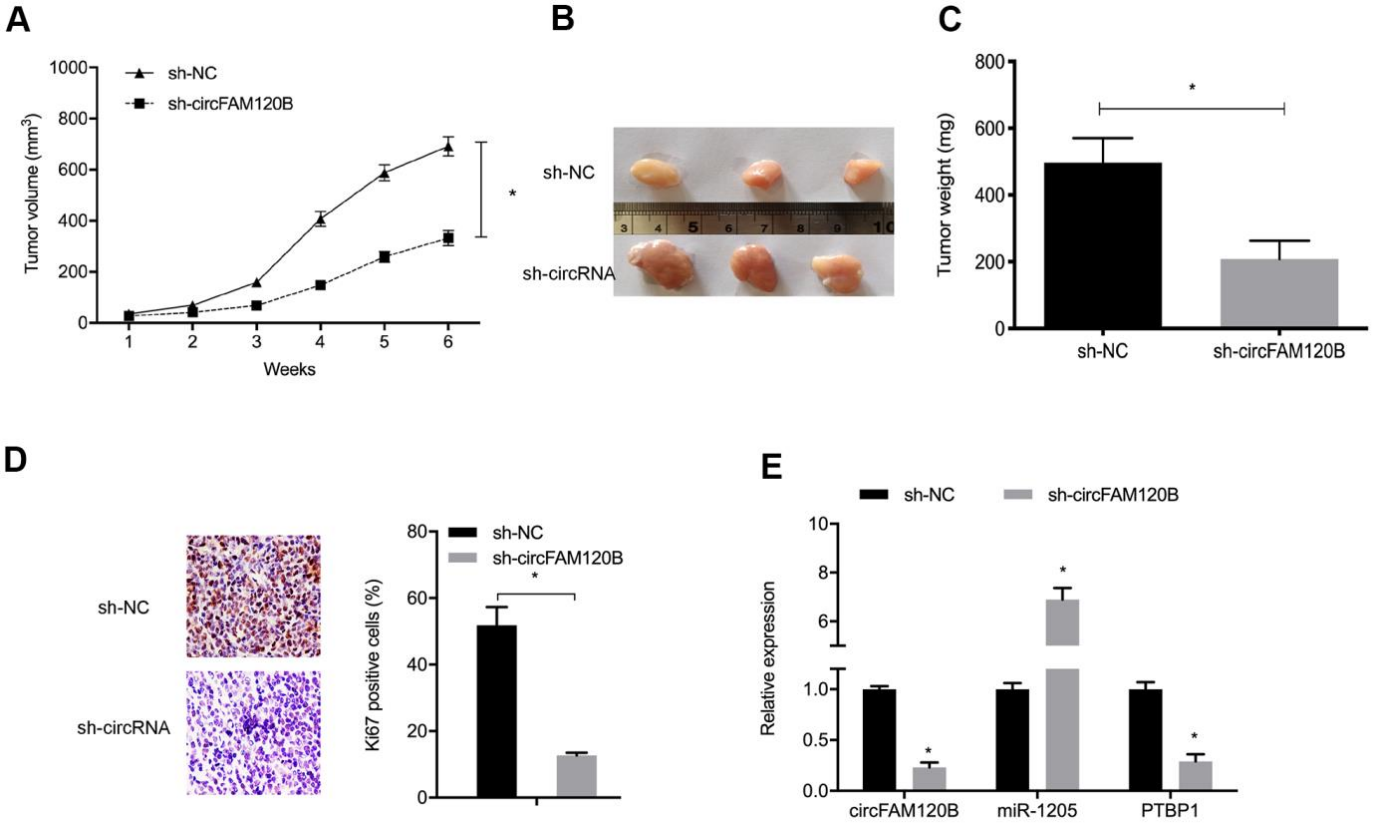
**circFAM120B suppression reduces *in vivo* tumor growth.** (**A**) Mouse tumor volumes were measured weekly. (**B**) Mouse tumor tissue images. (**C**) Mouse tumor weights. (**D**) Ki-67 expression in xenograft tissue. (**E**) CircFAM120B, miR-1205 and PTBP1 expression in xenograft tissue. *P < 0.05.

To comprehensively investigate circFAM120B functions during OS, an *in vivo* xenograft mouse model was established. Sh-circFAM120B decreased both tumor volume and tumor weight in mice (Figure 8A–8D). In excised tumors, expression data indicated that sh-circFAM120B reduced circFAM120B and PTBP1 levels but elevated miR-1205 levels when compared with the sh-NC group (Figure 8E). Combined, circFAM120B accelerated OS progression by modulating miR-1205/PTBP1 signaling.

## DISCUSSION

circRNAs are non-coding RNAs comprising a unique circular covalently bonded structure. They are promising molecular biomarkers in cancer due to their conserved, abundant, and tissue specific characteristics [5, 19]. Due to the development of high-throughput sequencing technologies, increasingly circRNAs have been implicated in OS. circECE1 activated energy metabolism in OS by stabilizing c-Myc [20]. Also, circNASP promoted key OS phenotypes by targeting miR-1253/FOXF1 signaling [21] and circTADA2A promoted cancer phenotypes in OS by sponging miR-203a-3p and modulating CREB3 [22]. In our study, circFAM120B was highly elevated in OS samples, and this was related to advanced TNM stage and metastasis. CircFAM120B knockdown suppressed OS cells proliferation, invasion *in vivo* and tumor growth *in vivo*, suggesting circFAM120B was associated with OS development.

Increasingly, studies have shown that circRNAs sponge miRNAs to impact miRNAs and subsequent gene expression [4, 17]. Therefore, to explore molecular mechanisms whereby circFAM120B putatively promoted OS progression, we firstly localized circFAM120B to the cytoplasm. Next, we identified miR-1205 as a downstream target of circFAM120B. To ascertain if miR-1205 was correlated to circFAM120B in regulating OS processes, we performed rescue assays and showed circFAM120B functioned in OS cells by sponging miR-1205.

Polypyrimidine tract-binding protein 1 (PTBP1) is reported as a tumor promoter in some cancers [23, 24]. PTBP1 promoted breast cancer cell growth by regulating PTEN/Akt signaling and autophagy [25]. Also, miR-194 inhibited key liver cancer phenotypes by modulating PTBP1/CCND3 signaling [26]. Similarly, circGLIS3 promoted non-small cell lung cancer phenotypes by affecting miR-644a/PTBP1 signaling [27].

In our study, PTBP1 levels were augmented in OS samples whereas PTBP1 silencing abrogated OS proliferation and invasion. Similarly, PTBP1 was identified as a candidate target gene for miR-1205; overexpressed miR-1205 reduced both PTBP1 mRNA and protein levels. Moreover, rescue assays showed PTBP1 overexpression abolished the effects of circFAM120B suppression on OS cells proliferation and invasion. Together, circFAM120B functioned as a competing endogenous RNA (ceRNA) promoting OS phenotypes by regulating miR-1205/PTBP1 signaling.

## CONCLUSIONS

circFAM120B exerts pro-cancerous functions during OS progression by protecting PTBP1 via miR-1205 sponging. By identifying these roles in OS and confirming a mechanism for circFAM120B, we provide valuable molecular targets for OS treatment.
